# Stability of soil slope in Almaty covered with steel slag under the effect of rainfall

**DOI:** 10.1038/s41598-024-58364-5

**Published:** 2024-04-02

**Authors:** Rezat Abishev, Alfrendo Satyanaga, Gulnur Pernebekova, Harianto Rahardjo, Qian Zhai, Chang-Seon Shon, Sung-Woo Moon, Jong Kim

**Affiliations:** 1 Department of Civil and Environmental Enginering, School of Engineering and Digital Sciences, Nazarbayev University, 53 Kabanbay Batyr Ave, 010000 Astana, Kazakhstan; 2 School of Civil and Environmental Engineering, Nanyang Technological University, 50 Nanyang Avenue, Singapore, Singapore; 3 Key Laboratory of Concrete and Prestressed Concrete Structures of Ministry of Education, School of Civil Engineering, Southeast University, Nanjing, 210096 China

**Keywords:** Rainfall, Unsaturated soil, Slope stability, Steel slag, Civil engineering, Hydrogeology

## Abstract

The issue of rainfall-induced slope failure has attracted more attention from geotechnical engineers as a consequence of global warming. Current cumulative waste disposal has generated scientific interest in the utilization of waste materials in geotechnical design for climate change adaptation measures. Taking into consideration the effect of slope height and angle, steel slag—a waste product derived from the production of steel—was investigated as a slope cover against rainfall. To assess the stability of the slope and the infiltration of water into the soil, numerical analyses were conducted using both SEEP/W and SLOPE/W software in conjunction with rainfall conditions. Based on the findings, it can be concluded that increasing the slope's elevation and inclination will have an adverse effect on its safety factor. Steel slag can nevertheless be utilized for minimizing rainwater infiltration into the slope, as indicated by the pore-water pressure variations and graphs of the safety factor versus time. For a 20-m slope height, steel slag slopes have demonstrated a lower factor of safety difference in comparison to the initial slope without remediation. Regardless of slope angle and slope height, the safety factor reduces marginally during rainfall.

## Introduction

Climate change entails the rise of severe weather conditions, such as increased rainfall intensity or its prolonged absence in the instance of wildfires^1,2^. As a result of frequent cycles of drought, rainfall, and heatwaves, climate change causes severe desiccation cracks^3^. The structural strength of clayey soils that are subjected to cyclic variations in water content is compromised by these desiccation cracks^4^. The prediction of thermal crack propagation in such soils is imprecise due to ambiguous field observations, modeling approximations, and underlying soil uncertainty^5^. One of the alternative ways to tackle these concerns is to develop machine learning models.

At the same time, waste generation and dumping are considered significant contributors to global climate change^6^. Cities, hotbeds of dynamic industrial development and population growth, generate tons of waste daily that must be managed^7,8^. Low-effective policy, poor transportation services, and the absence of adequate waste treatment facilities contribute to inadequate solid waste management, resulting in water, soil, and air pollution and exposing people and the environment to danger^9^. Environmental and human health concerns and the influence of economic growth are all factors in the growing use of these wastes as sustainable materials across the world^10^. Steel slag, which is a by-product produced in the steel industry, is one of those waste materials that attracts the attention of many researchers around the world with its possible application in industry for the promotion of sustainable development^11,12^. Steel is used in the construction of many structures across the world, which is why it is critical to recycle steel for various purposes, such as slope stabilization systems.

Numerous factors, such as hydrological processes, temperature variations, volcanism, seismic activity, and anthropogenic drivers, affect the stability of slopes^13,14^. In general, hydrological processes like precipitation, infiltration, runoff, and evapotranspiration have a massive effect on the initiation of a landslide, which is the movement of a mass of rock, rubble, or soil down natural or anthropogenic slopes^15,16^. Among all these processes, rainfall is eminently the most common cause of landslides, as it influences most hydrological processes. Infiltration of rainwater creates transient changes in groundwater dynamics, decreasing slope stability^10,17^. Owing to the rise in pore water pressure, the material's effective shear strength drops, eventually falling below a given threshold, and this triggers the movement of the slope^18,19^. The combination of infiltration and runoff, depending on the intensity and frequency of the rainfall, can lead to all sorts of mass movements at various depths^10,20^. Rainfall-induced landslides are a major threat to populations and infrastructure. Nguyen et al.^21^ mention in their case study work that rainfall-induced landslides are a major threat to populations and infrastructure. Hence, researchers examined rainfall data for a sandstone slope in Japan, considering the intensity, duration, and frequency of rainfall events. The data was analysed in relation to landslide occurrences to determine the rainfall thresholds that lead to landslides.

Preventive actions are required to protect the slope against the threat of rainfall-induced slope failures, ensuring the protection of neighbouring structures or community facilities. It is important to find cost-efficient and sustainable alternatives to ensure slope stability. According to Rahardjo et al.^22^ subsurface drainage (horizontal drains), capillary barrier system, and the use of vegetation are all known methods for landslide stabilization, which naturally imply slope stability. Nguyen et al.^23^ suggest in their study that by incorporating vegetation-based approaches into slope management, the risk of landslides can be minimised and slope stability can be enhanced. In the meantime, Ongpaporn et al.^24^ revealed the significance of bio-engineering as a sustainable method for slope stabilization and erosion control in environmentally sensitive regions, like Southern Thailand. Another potential way to protect the slope from rainfall is to use waste material. Instead of natural materials, like sand or gravel, which are limited in certain countries, waste materials can be recycled for slope stabilization. This paper investigates the feasibility of steel slag as a slope cover to prevent slope failure with different slope heights and angles, since no research has been conducted on the use of steel slag to maintain slope stability during rainfall in Kazakhstan, incorporating unsaturated soil mechanics.

Many research and construction projects worldwide have recently focused on unsaturated soils, as many soils near the ground surface are deemed unsaturated^25,26^. Unsaturated soil is observed within the soil above the water table, where pore-water pressure is negative^27^. Negative pore-water pressure is usually referred to as matric suction, a central aspect of unsaturated soil mechanics^28^. In contrast to saturated soil, apart from local interparticle forces that include physicochemical forces, surface tension exists at air–water interfaces due to the aforementioned matric suction^29^. The soil–water characteristic curve (SWCC) describes the relationship between matric suction and volumetric water content^30,31^. SWCC is used in many models to estimate the shear strength of unsaturated soil^32,33^. SWCC and unsaturated permeability are necessary for conducting finite element analyses to understand the changes in matric suction due to rainfall and the dry effect^34,35^. In addition, unsaturated shear strength is an essential parameter for performing slope stability analyses under dry and rainy periods^36^. Nguyen and Likitlersuang^37^ highlight the importance of understanding the stability of unsaturated soil slopes, especially under rainfall conditions. According to them, due to fluctuations in water content, unsaturated soil slopes are susceptible to instability; therefore, evaluating their reliability is critical for mitigating risks, considering both hydraulic and shear strength parameters.

Brand et al.^38^ introduced the presence of a continual issue in terms of landslides in Hong Kong. Heavy rainstorms triggered more than 90% of landslides in Hong Kong^38^. Thurman^39^ presented a broad overview of catastrophe effects in Central Asia, reporting that landslides are widespread in Central Asia's mountainous regions. Increased slope steepness (due to geological processes), seismic occurrences, meteorological and hydrological abnormalities, and a range of human causal factors, all induce landslides in that area. Komolvilas et al.^40^ also emphasize the importance of considering all the above-mentioned factors in landslide risk management by introducing the occurrence of a landslide event in Huay Khab Mountain. Based on long-term data, in Uzbekistan, scientists have discovered an essential link between landslide activation and 4- to 5-year cycles of rainy and dry years^39^. As Kazakhstan is a part of Central Asia, the landslide pattern would be almost the same as it was presented^39^. Accelerated glacier melting or heavy rainfall events can induce landslides and mudslides. In mountainous areas, heavy rainfall events and rapid melting glaciers destabilize mountain slopes, resulting in landslides. Extreme rainfall occurrences are the primary cause of mudslides in Kazakhstan. Mudslides, which are a form of landslide, are especially prevalent in mountain rivers. In 2013, one of these disasters occurred on the Sarysay River as a result of extreme precipitation^41^. Mudslides resulting in substantial damage were only recorded in the Almaty oblast between 1967 and 1990^41^. Generally, according to Chepelianskaia and Sarkar-Swaisgood^42^, the Southeast and East regions of Kazakhstan are at high and medium risks of landslides, respectively. Considering the presence of landslide hazards in the Almaty region, there is a need to introduce sustainable mitigation measures.

Researchers discovered that steel slag could be used for several construction purposes, such as concrete, road subgrade, and railway ballast materials, due to its high strength^43,44^. The reason behind the use of steel slag in road construction is that its properties are suited to unbound and asphalt layers^45^. Owing to its high strength and durability, the application of steel slag in road construction was credible, as it could produce high-quality aggregates equivalent to natural aggregates. Furthermore, properties like high abrasion and its rough texture made it suitable for hydraulic construction^46^. Yi et al.^46^ mentioned in their report that since 1993, the Nippon Slag Association in Japan had been researching employing steelmaking slag as a material for ground enhancement in constructing ports and harbours. Despite the fact that steel slag can enhance engineering properties, its chemical composition can pose a threat to the environment. Leelarungroj et al.^47^ have studied the leaching mechanisms of heavy metals from cement and fly ash stabilised soils. Common heavy metals found on site consist of arsenic, chromium, lead, and zinc^47,48^. Since fly ash and steel slag are both commonly used industrial byproducts in a wide range of construction and engineering applications, it is assumed that both have similarities in being potentially hazardous waste materials because of heavy metals and other contaminants. These contaminants may be released when the materials are exposed to water, which can create potential environmental risks. To avoid the leaching concern, phytoremediation could be an effective approach to remediation^48^. According to the literature, steel slag is widely used in concrete research and pavement design. However, there is little research on the application of steel slag in a geotechnical domain, namely slope stabilization, in Kazakhstan, particularly using unsaturated soil mechanics. Therefore, this study aims to evaluate the performance of steel slag in a slope cover system for typical slope geometries observed in the Almaty region.

## Materials and methods

### Soil properties used in the analyses

**Figure 1:**
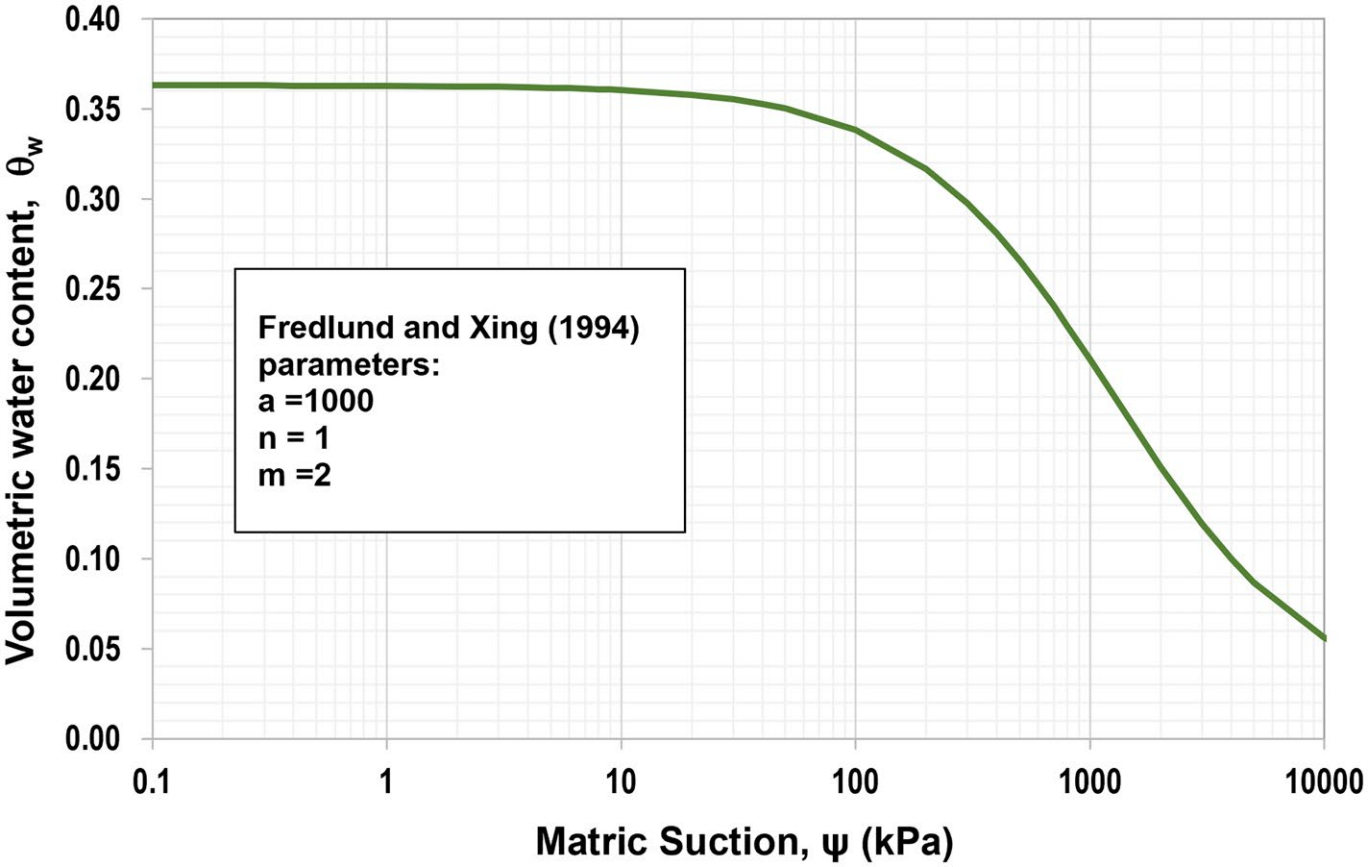
SWCC of the original soil.

**Figure 2:**
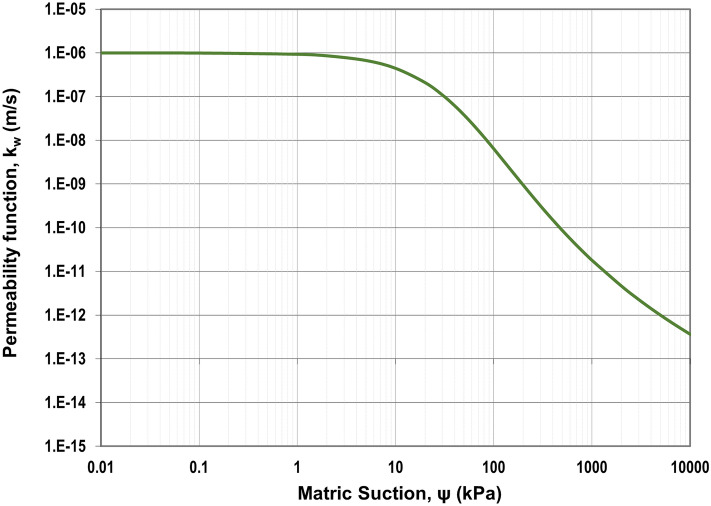
Unsaturated permeability of the original soil.

This study was conducted on the typical slope in Almaty since many historical slope failures happened in this region. Considering the fact that there are no available samples of an original soil to be examined, the known parameters of one of the native soils from the Almaty region were taken from the study of Sharipov et al.^49^. Therefore, to assign the shear strength properties of the original soil, the properties of a clayey loam with 5% fine pebbles were used as the material properties in the model: unit weight (γ) = 17 kN/m^3^, ϕ′ = 19º, and c′ = 33 kPa^49^. The hydraulic properties of an unsaturated soil (SWCC and unsaturated permeability) were estimated using the USCS soil type according to Mercer et al.^50^ guidelines. In other words, the SWCC curve was created using parameters whose values were derived from predictions made using the USCS classification and can be found in Fig. 1. Figure 2 presents the permeability function of the original soil determined from SWCC and saturated permeability using the statistical method following the procedure explained by Satyanaga et al.^51^. Both SWCC and the permeability function of the original soil were included in the seepage analyses.

### Applicable theories

SWCC is vital to developing unsaturated soils, and many empirical equations have been proposed to best fit or predict SWCC. One of the equations is proposed by Satyanaga et al.^52^ as follows:

1 $${{\theta }_{w}}_{ }=\left(1-\frac{{\text{ln}}\left(1+\frac{\psi }{{\psi }_{r}}\right)}{{\text{ln}}\left(1+\frac{{10}^{6}}{{\psi }_{r}}\right)}\right)\times [{\theta }_{r}+\left\{\left({\theta }_{s}-{\theta }_{r}\right)\left(1-\left(\beta \right)erfc\left(\frac{\mathit{ln}\left(\frac{{\psi }_{a}-\psi }{{\psi }_{a}-{\psi }_{m}}\right)}{s}\right)\right)\right\}]$$

where: β = 0 when $\psi$ ≤ ${\psi }_{a}$; β = 1 when $\psi$ > ${\psi }_{a}$; ${{\theta }_{w}}$= calculated volumetric water content; ${\theta }_{s}$ = saturated volumetric water content;$\psi$ = matric suction under consideration (kPa); ${\psi }_{a}$ = parameter representing the air-entry value of the soil (kPa); ${\psi }_{m}$ = parameter representing the matric suction at the inflection point of the SWCC (kPa); ${\psi }_{r}$ = suction corresponding to the residual water content, ${\theta }_{r}$; s = parameter representing the geometric standard deviation of the SWCC;${\theta }_{r}$ = parameter representing the residual volumetric water content.

Water flows through a fully saturated soil in accordance with Darcy's empirical law^53^:

2 $$q=Aki$$

where: $q$ = volume of water flowing per unit time (m^3^/s); $A$ = the cross-sectional area of the soil corresponding to the flow q (m^2^); $k$ = coefficient of permeability with respect to the water phase (m/s); $i$ = hydraulic head gradient.

The coefficient of permeability is relatively constant in saturated soil. Darcy's law, applied to saturated soil, is also implemented for analyses of the flow of water in unsaturated soil. However, the coefficient of permeability of unsaturated soil cannot be assumed to be a constant, as it is a function of the water content or the matric suction in the unsaturated soil^30^.

As direct measurements of the water coefficient of permeability of unsaturated soil are usually difficult to perform, attempts to predict the coefficient of permeability are commonly done theoretically. These predictions are referred to as an indirect method to determine permeability. One way to predict the permeability coefficient is by using SWCC^30^. SWCC can be used to compute ${k}_{w}$ using Eq. (3):

3 $$k_{w} \left( \theta \right)_{i} = \frac{{k_{s} }}{{k_{sc} }}A_{d} \mathop \sum \limits_{j = i}^{m} \left[ {\left( {2j + 1 - 2i} \right)\left( {u_{a} - u_{w} } \right)_{j}^{ - 2} } \right],\quad i = 1,2, \ldots ,m$$

where: ${k}_{w}{(\theta )}_{i}$ = computed permeability coefficient at the $i$ th interval for a certain volumetric water content ${\theta }_{i}$ (m/s); $i$ = interval number that decreases with increasing water content (e.g., on the experimental SWCC, $i=m$ indicates the final interval that closely matches the lowest volumetric water content (${\theta }_{L}$), and $i=1$ indicates the initial interval that closely matches the saturated water volumetric content (${\theta }_{s}$)); ${A}_{d}$ = adjustment factor [i.e., (${{T}_{s}}^{2}{\rho }_{w}g/2{v}_{w}$)(${{\theta }_{s}}^{p}$/${N}^{2}$) (m ${{\text{s}}}^{-1}\cdot {\mathrm{ kPa}}^{2}$)]; ${k}_{sc}$ = computed saturated permeability coefficient (m/s); ${T}_{s}$ = water's surface tension (kN/m); ${\rho }_{w}$ = water density (kg/m^3^); $g$ = acceleration of gravity (m/s^2^); ${v}_{w}$ = water’s absolute viscosity (Ns/m^2^); $p$ = constant that addresses the interaction of pores of different diameters; $m$ = total number of intervals between ${\theta }_{s}$ and ${\theta }_{L}$ on the experimental SWCC; $N$ = total number of intervals calculated between ${\theta }_{s}$ and zero water content, i.e., $\theta$ = 0 (note: $N$ = $m$[${\theta }_{s}$/${(\theta }_{s}-{\theta }_{L})$], $m$ ≤ $N$, and $m$ = $N$ when ${\theta }_{L}$ = $0$).

Statistical hydraulic conductivity models may be used to indirectly predict the hydraulic conductivity function from measurements of the SWCC. This method is a slight modification of the Kunze et al.^54^ model so that SI units and matric suction can be used instead of a pore-water pressure head^55^. The calculations are done by dividing the relation between volumetric water content and matric suction into "m" equal water-content increments. The permeability coefficient is calculated using the matric suction corresponding to the midpoint of each interval.

The shear strength of unsaturated soil is expressed in terms of two independent stress state variables, namely the net normal stress $({\sigma }_{n}-{u}_{a}$) and the matric suction $({u}_{a}-{u}_{w}$) that represent the shear strength of unsaturated soil^56^. When an unsaturated soil approaches saturation, the pore-water pressure ${u}_{w}$ approaches the pore-air pressure ${u}_{a}$, and as a result, the matric suction $({u}_{a}-{u}_{w}$) will become zero. It will give the shear strength equation for saturated soil as proposed by Terzaghi^57^:

4 $$\tau ={{\text{c}}}^{\mathrm{^{\prime}}}+ {\left(\sigma -{u}_{w}\right)tan\phi }{\prime}$$

where: $\tau$ is the shear strength of the unsaturated soil (kPa); $c{\prime}$ is the effective cohesion of the soil (kPa); $({\sigma }_{n}-{u}_{w}$) is the effective stress on the failure plane at failure (kPa); ${\phi }{\prime}$ is the effective angle of internal friction (^o^).

A formula to describe the shear strength of unsaturated soil was proposed by Fredlund et al.^56^ and it was improved by Zhai et al.^58^ to allow the nonlinearity of ϕ^b^ angle with respect to suction. Zhai et al.^59^ simplified the shear strength function proposed by Zhai et al.^58^ for the unsaturated soil as shown in Eq. (5). In Eq. (5), the SWCC is fully incorporated into the shear strength function for the unsaturated soil.

5 $$\tau =c\mathrm{^{\prime}}+\left(\sigma -{u}_{a}\right)\mathit{tan}\phi \mathrm{^{\prime}}+\frac{S-S\mathrm{^{\prime}}}{1-S\mathrm{^{\prime}}}\left({u}_{a}-{u}_{w}\right)\mathit{tan}\phi \mathrm{^{\prime}}$$

where: S is the degree of saturation; S′ is the degree of saturation corresponding to the suction of 3100 kPa, $({\sigma }_{n}-{u}_{a}$) is the net normal stress on the failure plane at failure (kPa); $({u}_{a}-{u}_{w}$) is the matric suction at the point of failure (kPa).

By computing the governing partial differential equation of seepage, water seepage through saturated–unsaturated soil can be studied in both its steady and transient states. The solution is found by employing numerical modelling methods such as finite difference and finite element methodologies^60^. To solve two-dimensional transient seepage problems, the finite element method is employed, incorporating Eqs. (6) and (7) for saturated and unsaturated soils, respectively. Unsteady state seepage equations:

6 $${\text{Saturated}}:\;\;k_{s} \left( {\frac{{\partial^{2} h_{w} }}{{\partial x^{2} }}} \right) + k_{s} \frac{\partial }{\partial y}\left( {\frac{{\partial^{2} h_{w} }}{{\partial y^{2} }}} \right) = { }m_{v} \rho_{w} g\frac{{\partial h_{w} }}{\partial t}$$

7 $${\text{Unsaturated}}:\;\frac{\partial }{\partial x}\left( {k_{w} \frac{{\partial h_{w} }}{\partial x}} \right) + \frac{\partial }{\partial y}\left( {k_{w} \frac{{\partial h_{w} }}{\partial y}} \right) = { }m_{2}^{w} \rho_{w} g\frac{{\partial h_{w} }}{\partial t}$$

Slope stability analysis is based on limit equilibrium, and there are two distinct factors of safety (FOS) equations: one for horizontal force equilibrium and the other for moment equilibrium^60^. On the assumption that soil pore-air pressure is atmospheric, i.e., ${u}_{a}$ = 0, the FOS equations with respect to force equilibrium for saturated and unsaturated soils are as follows:

8 $${\text{Saturated:}}\;F_{f} = \frac{{\sum \left[ {c^{\prime}\beta \cos \alpha + \left\{ {N - u_{w} \beta } \right\}\tan \varphi^{\prime}\cos \alpha } \right]}}{\sum N\sin \alpha }$$

9 $${\text{Unsaturated}}:\;F_{f} = \frac{{\sum \left[ {c^{\prime}\beta \cos \alpha + \left\{ {N - u_{w} \beta \frac{{\tan \varphi^{b} }}{\tan \varphi ^{\prime}}} \right\}\tan \varphi^{\prime}\cos \alpha } \right]}}{\sum N\sin \alpha }$$

where: ${F}_{f}$ = FOS with respect to force equilibrium.

In the case of moment equilibrium, considering zero pore-air pressure, FOS equations for saturated and unsaturated conditions are presented in the following forms:

10 $${\text{Saturated}}:F_{m} = \frac{{\sum \left[ {c^{\prime}\beta R + \left\{ {N - u_{w} \beta } \right\}R\tan \varphi^{\prime}} \right]}}{\sum Wx - \sum Nf}$$

11 $${\text{Unsaturated}}:\;F_{m} = \frac{{\sum \left[ {c^{\prime}\beta R + \left\{ {N - u_{w} \beta \frac{{\tan \varphi^{b} }}{\tan \varphi ^{\prime}}} \right\}R\tan \varphi^{\prime}} \right]}}{\sum Wx - \sum Nf}$$

where: ${F}_{m}$ = FOS with respect to moment equilibrium.

### Laboratory Testing

The laboratory testing included index property tests and SWCC tests. The test specimens had a height of 50 mm. The specimens were compacted based on the density of steel slag from the study by Rahardjo et al.^61^ in five layers, each of which had a thickness of 10 mm. The index property tests consisted of specific gravity and GSD tests conducted in accordance with the testing standards. The specimen's grain size analysis was conducted per ASTM D422-63R07^62^. Following ASTM D854-14^63^, specific gravity test was performed using a water pycnometer. The saturated coefficients of permeability were determined from the constant head permeability test using a triaxial permeameter with two back pressure systems^64^. A constant head triaxial permeability test is the best way to determine slow flow rates. The test can be done with a wide range of hydraulic gradients and effective confining stress, as well as pore pressures that are relevant to the site conditions.

The triaxial test for determining the specimen permeability consisted of three stages: saturation, consolidation, and permeability. The saturated permeability test applied water pressure to the specimen under a particular cell and back pressure. Due to the water pressure applied, a pressure difference was created, and water would flow when the drainage inlet was opened. As the water flowed through the specimen, the volume change of water and the elapsed time were measured. The saturated permeability (k_s_) can then be calculated using Darcy's law, as shown in Eq. (2). The saturated permeability test was performed multiple times with different water pressures, and an average value of the ${k}_{s}$ was then calculated.

A consolidated-drained (CD) triaxial test is used to determine the effective cohesion (c') and effective friction angle (ϕ') of soils. It is a compression test in which the sample has been allowed to consolidate to its equilibrium moisture content during the application of the cell pressure before shear, and the drainage taps are opened during shearing, in which water is free to flow out of or into the test specimen^65^. Drainage is permitted throughout the test so that consolidation occurs completely under the all-around stress and no excess pore pressure develops during applying the deviator stress^66^.

The triaxial test is done by putting a cylindrical soil specimen enclosed in a rubber membrane on a triaxial cell. To set up the specimen for the test, a metal split mould is used to hold the specimen from falling at the initial state. The mould has an internal diameter of 7 cm. A special rubber membrane of thickness 1 mm with two O rings securely attached to the base of the triaxial cell is enclosed in the mould. A porous stone and filter paper are inserted inside the rubber membrane. The specimen is then continuously poured into the inner part of the rubber membrane, layer by layer. Each layer was tampered with 50 counts with a hammer. When the specimen is filled to a height of 140 mm, a vacuum is applied to the specimen, and then the metal split mould is removed. The vacuum is to help the specimen stand on its own without the mould. Next, put filter paper and a porous stone on the specimen. Next, the rubber membrane is secured with another two O rings at the top of the specimen. Finally, the vacuum pipe is removed, and the triaxial cell is filled with water.

Before consolidating a specimen in the triaxial cell, the degree of saturation needs to be checked. It is measured by the increase in pore water pressure Δ*u* in response to an increase in cell pressure Δσc with the drainage taps closed. If the specimen is fully saturated, the ratio Δ*u*/Δσc (known as the B-value) should equal one. A B-value of less than one indicates that air is present in the soil pores, and the back pressure should be increased to dissolve it^65^.

**Figure 3:**
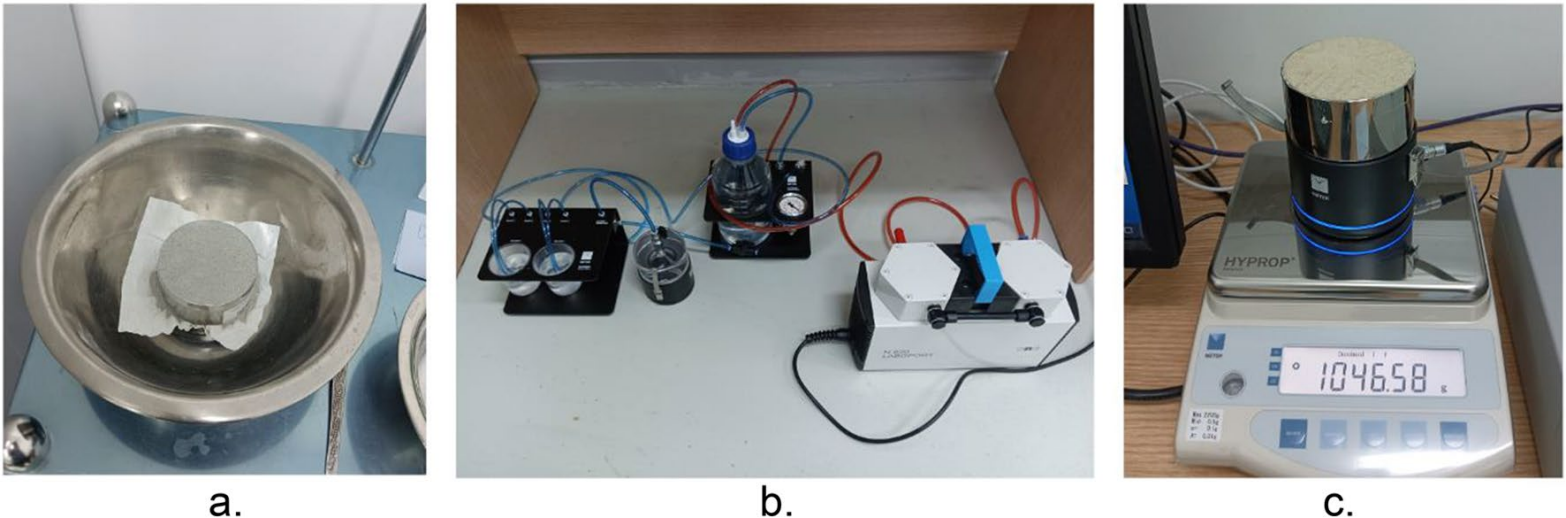
(**a**) Saturation of the soil specimen, (**b**) saturation of tensiometers, (**c**) sensor unit with a soil sample on the balance.

There are several ways to measure SWCC: using Tempe Cell Test and Pressure Plate Equipment or using HYPROP Equipment. The latter is the more accurate and, more importantly, faster approach to finding SWCC. It is the process that relies on Wind's (1966) evaporation approach, simplified by Schindler's (1980) model^67,68^. In this work, SWCC was measured while operating with HYPROP equipment. Figure 3 shows the saturation of the soil sample and tensiometers, while the general procedure involves the following steps:

- soil sampling and saturation of the soil specimen.
- preparation of the measuring equipment.
- setting up the specimen in the device.
- start of measurement.
- analysis of the obtained data with HYPROP-FIT.

The material was prepared in accordance with the volume of a sample ring and compacted in 5 layers for the overall 5 cm height of the sample ring, with each 1 cm layer compacted by 25 blows^69^. After compaction, the specimen was saturated by immersing the sample ring with the material in a bowl of water. Meanwhile, tension shafts or tensiometers with ceramic tips were placed in the beaker with deionized water. After the saturation process, using the tension shaft auger, holes were drilled inside the soil. To ensure that air would not be forced into the soil sample during assembly, drill holes were filled with water. The sensor unit's holes were filled bubble-free using a droplet syringe, and following that, tensiometers were screwed into the openings. The entire test assembly was turned upside down after carefully placing the sensor unit on the soil sample. The next step was to remove the saturation plate and nonwoven cloth, then delicately clean and dry the sample ring and clips. Following this preparation of the soil sample, measurement had been started^67^.

The complete relationship between volumetric water content and soil suction was obtained by best-fitting the HYPROP test results using the unimodal fitting equation developed by Satyanaga et al.^52^.

### Waste material used in the analyses

**Figure 4:**
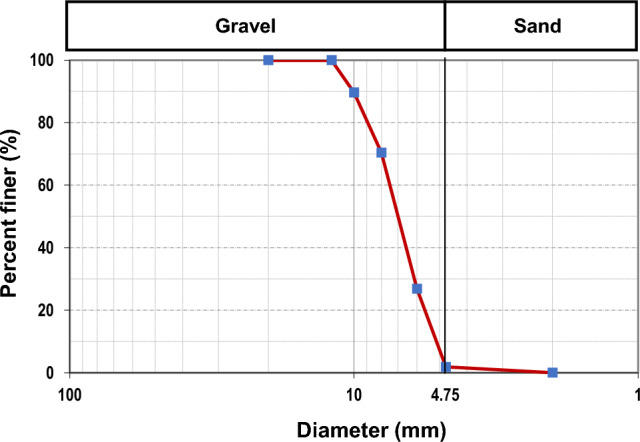
Particle size distribution of steel slag.

For a slope stabilization system, steel slag was introduced as a slope cover. Table 1 lists the index properties of steel slag, and Fig. 4 shows its grain size distribution curve. Based on the grain size analysis, steel slag appeared to have 98.11% of gravel content larger than 4.75 mm.

**Table 1:** Index properties of steel slag.

	Steel Slag
Specific gravity, G_s_	3.50
Porosity, n	0.27
Dry density, ρ_d_ (Mg/m^3^)	1.54
Gravel content,%	
(More than 4.75 mm)	98.11
Sand content, %	1.89
Fines content,%	
(Less than 0.075 mm)	0

Adhering to the guidelines in a HYPROP manual and based on the evaporation method, SWCC of steel slag was generated. Equation (1) was used to best fit the experimental data of SWCC. The best-fitting parameters of Eq. (1) can be found in Table 2.

**Table 2:** Best fitting parameters of Satyanaga et al.^52^ equation.

SWCC parameter	Symbol	Value
Saturated Volumetric Water Content	θ_s_	0.270
Residual Volumetric Water Content	θ_r_	0
Suction at the air-entry value (kPa)	Ψ_AEV_	0.300
Suction at the inflection point (kPa)	Ψ_m_	1.034
Suction at the residual water content, ${\theta }_{r}$ (kPa)	Ψ_r_	10

**Figure 5:**
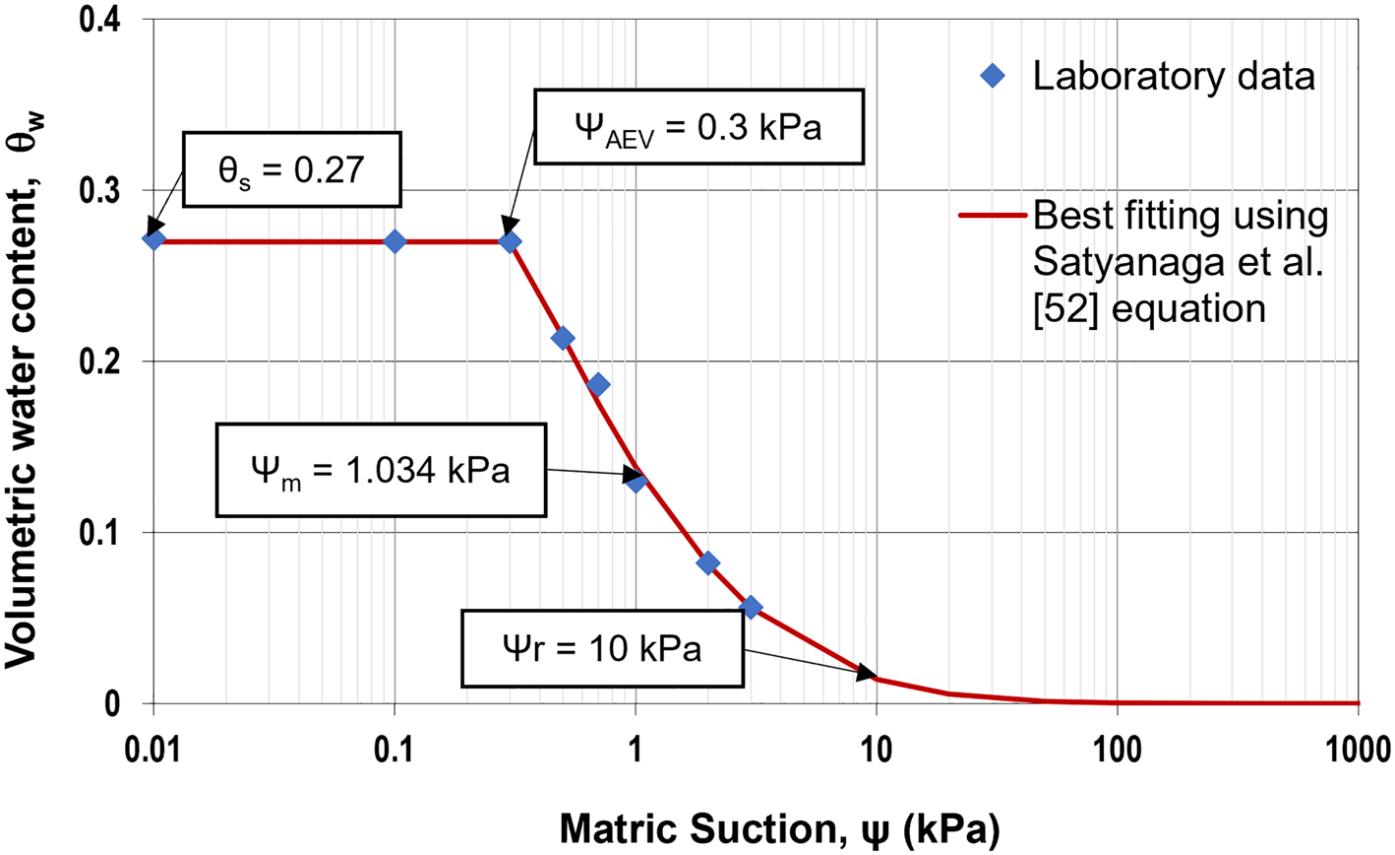
SWCC of steel slag.

To use Eq. (1) for modelling SWCC, each parameter must have a proper initial value^52^. To best fit the laboratory data of the SWCC, all the parameters can be adjusted via an iterative non-linear regression approach offered in the Microsoft Excel software^68,70^. Accordingly, the relationship between volumetric water content and matric suction can be observed in Fig. 5. Eventually, SWCC data were applied in subsequent seepage analyses.

**Figure 6:**
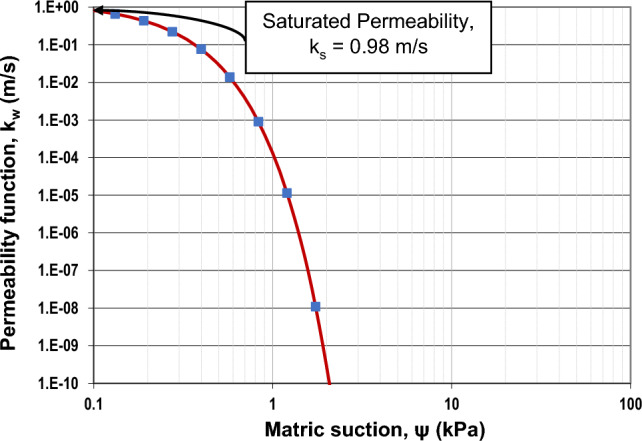
Unsaturated permeability of steel slag.

Using the statistical method described by Satyanaga et al.^51^, the permeability function of steel slag was obtained from the SWCC and the saturated permeability, as shown in Fig. 6. The permeability function was also incorporated in the seepage analyses together with the best-fit SWCC data.

**Figure 7:**
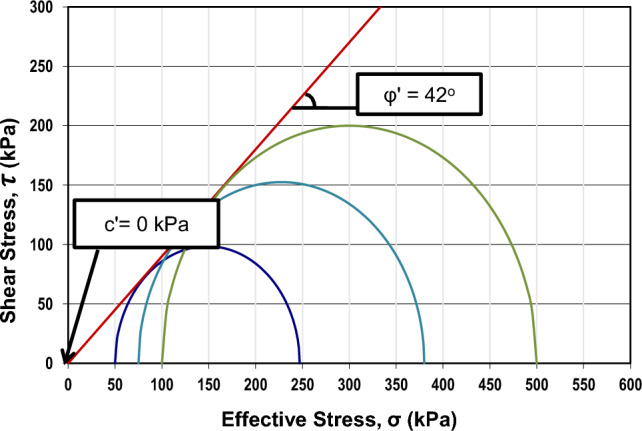
Mohr–Coulomb failure envelope of steel slag constructed from triaxial tests.

Following triaxial test results, the shear strength parameters of the steel slag were found as follows: c' = 0 kPa, γ = 18 kN/m^3^, and ϕ' = 42º, which can be seen in Fig. 7. The unsaturated shear strength angle (ϕ^b^) was 21º since ϕ^b^ was taken as half of ϕ'^71^.

According to Figs. 1 and 5, the saturated volumetric water content (θ_s_) of steel slag is 0.27, while the original has a higher value, which is equal to 0.36. Likewise, the air-entry value (Ψ_AEV_) of steel slag is 0.3 kPa, which is much lower than the air-entry value of original soil (Ψ_AEV_ = 150 kPa). It can be stated that coarser particles tend to have lower air-entry values than finer particles. Based on Figs. 2 and 6, the saturated permeability of steel slag is k_s_ = 0.98 m/s; in the meantime, for original soil, it is substantially lower, k_s_ = 1e-6 m/s. It is observed that steel slag has a much lower coefficient of permeability at high suction ranges in comparison to the original soil. With regards to shear strength parameters, steel slag is cohesionless soil, while original soil has an effective cohesion (c’) equal to 33 kPa. In addition, the effective friction angle (ϕ’) and the unsaturated shear strength angle (ϕ^b^) are greater for steel slag.

### Numerical analyses

The numerical analyses were performed using GeoStudio software, particularly SEEP/W for seepage analysis and SLOPE/W for slope stability analysis. GeoStudio is integrated software that can analyse the stability and seepage of soil slopes. The numerical analysis results can be used to verify the findings from laboratory experiments and field studies^72,73^. SEEP/W, which employs a finite element method for seepage analyses, and SLOPE/W, which is based on the limit equilibrium method for stability analyses, are GeoStudio software products that many researchers commonly use. In the research by Yeh and Tsai^74^, slopes with uniform soil were put through two-dimensional transient seepage analyses using the SEEP/W module to see how changes in the amount of rain in 2016, 2050, and 2100 would affect the slope's hydrological state. Afterwards, the results of the seepage analyses were input into the SLOPE/W module to compute the slope safety coefficient. Yu et al.^73^ analyzed a homogenous slope in Luogang District, Guangzhou City, China, utilizing the SEEP/W and SLOPE/W modules. Yuan et al.^75^ developed the multilevel-filled soil slope seepage model using SEEP/W, examined the surface infiltration law, and investigated the slope's stability with SLOPE/W. Bračko et al.^76^ carried out the stability analysis using the SEEP/W module for the slope's surface infiltration model, studying rainwater infiltration to assess the impact of climate change on slope stability. Srikanth et al.^77^ examined the factors affecting the stability of a homogeneous unsaturated pond ash slope employing the finite element and limit equilibrium methods contained in GeoStudio. Although GeoStudio is widely utilized in slope stability analyses under rainfall conditions among researchers, it has some limitations. In the current version of SEEP/W, one type of SWCC, either drying or wetting, can be incorporated in the seepage analyses. At the moment, it is not feasible to have both. Another limitation is that for slope stability analyses, only one ϕ^b^ value is used; however, it should change for different suctions.

Following the study by Sharipov et al.^49^, in this research, thirteen sets of analyses were conducted for slope heights of 10 m, 20 m, and 30 m and slope angles of 27°, 45°, and 70°, where four of the analyses were examined without steel slag to confirm its effectiveness. A study by Sharipov et al.^49^ indicated that slope failure was observed within 3 m of the ground surface in Almaty. Therefore, the thickness of steel slag was taken as 3 m from the ground surface to be used as the slope cover. The extension of 3 m at the crest of the slope was also adopted in the analysis based on the previous study^78^. In Kazakhstan, it is common to have 3 m of soil replaced for slope rectification measures. Since the rainiest period takes 12 days^49^, the duration of each analysis was assigned as 24 days, where the first half was subjected to rainfall; it is referred to as a wet period. It was decided to make the remaining half of the duration a dry period to additionally check the drying effect.

**Figure 8:**
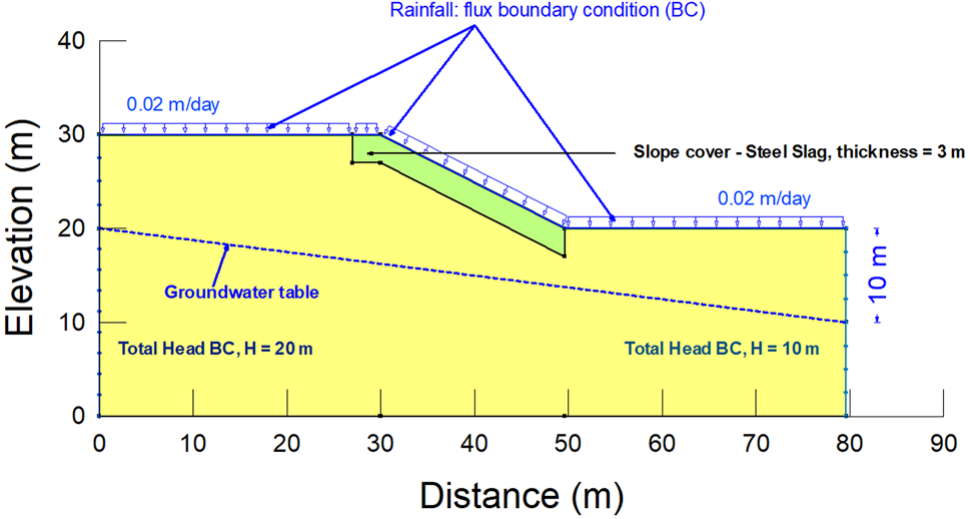
A numerical model for seepage analyses with a slope height of 10 m and slope angle of a 27°

Before creating the numerical model for seepage analysis in SEEP/W and SLOPE/W, the drawing sketch was prepared to identify the coordinates for convenience. Afterwards, a numerical model was built in GeoStudio. Accordingly, the scale was adjusted, and the axes were drawn. When choosing a material model, a saturated/unsaturated scenario is considered. In this case, computed unsaturated permeability functions and SWCCs of the original soil and steel slag were inserted into hydraulic conductivity and volumetric water content functions, respectively. The groundwater table is found 3–10 m below the ground surface with respect to the local data in Almaty^79^. Therefore, the groundwater table was placed 10 m below the ground surface. The maximum daily rainfall of 20 mm in Kazakhstan was adopted in the numerical analyses^42^. Rainfall intensity was assigned to 20 mm/day on the model's surface, and rainfall was applied as a flux boundary condition. Other assigned boundary conditions are constant total heads (left and right), the values of which depend on the location of the groundwater table. This boundary condition was assigned to avoid the effect of the side boundary condition on the investigated slope. The boundary conditions play a crucial role in the seepage analysis since the results of the numerical analysis are contingent upon the boundary conditions and input parameters^80,81^. Figure 8 illustrates a numerical model for seepage analyses of the slope with 10 m height and 27° inclination.

**Figure 9:**
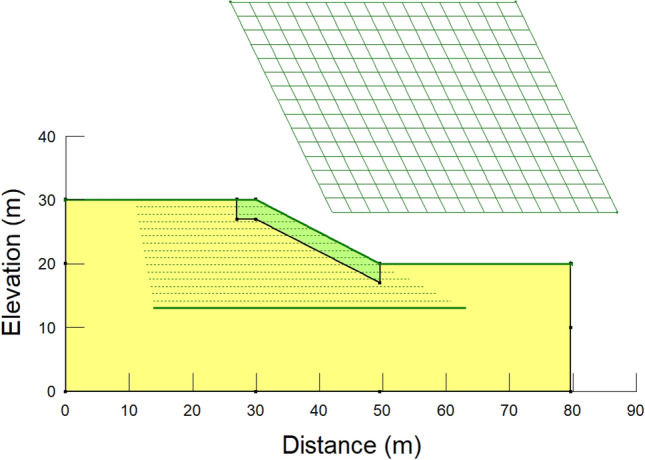
A numerical model for slope stability analyses with a slope height of 10 m and slope angle of a 27^o^.

The pore-water pressure variations from seepage analyses were incorporated into the slope stability analyses. The limit equilibrium method was utilized to conduct slope stability analyses. The Morgenstern-Price method was chosen to determine the safety factor as it satisfies both force and moment equilibrium. The analysis was conducted by setting the material model as Mohr–Coulomb and correspondingly inputting shear strength parameters. The grid and radius method was used in SLOPE/W for the slip surface, and both were constructed by trial and error, as demonstrated in Fig. 9. The critical value of factors of safety was determined by taking the minimum values of safety factors identified at each centre^82^. Hence, since it was found in the centre of the grid, the result of a factor of safety was assumed to be accurate.

## Results and discussion

**Figure 10:**
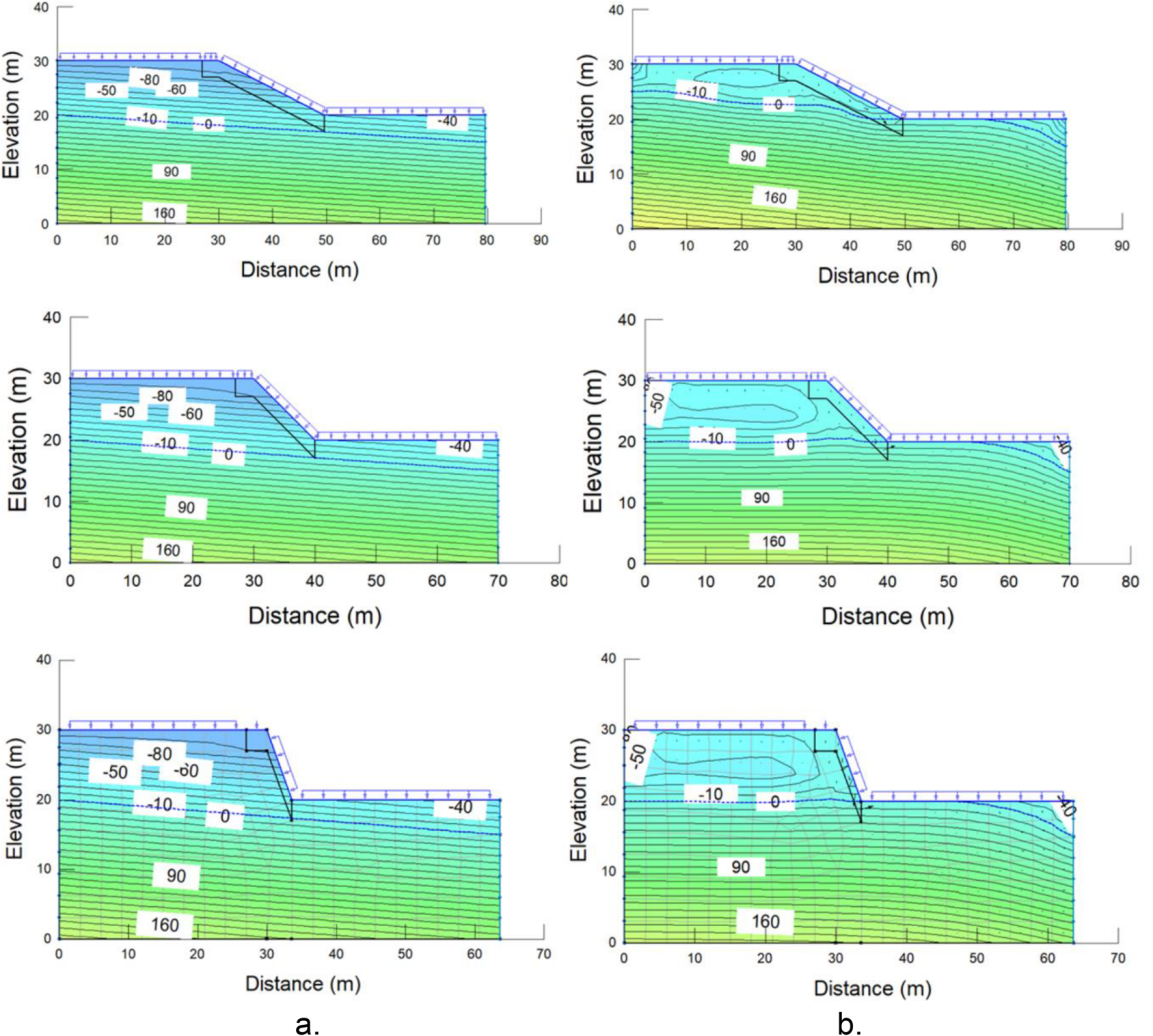
Pore-water pressure contours (**a**) at t = 0 day (before rainfall started) and (**b**) at t = 12 day (at the end of rainfall) for slope height = 10 m and angles = 27° (top); 45° (middle); 70° (bottom).

**Figure 11:**
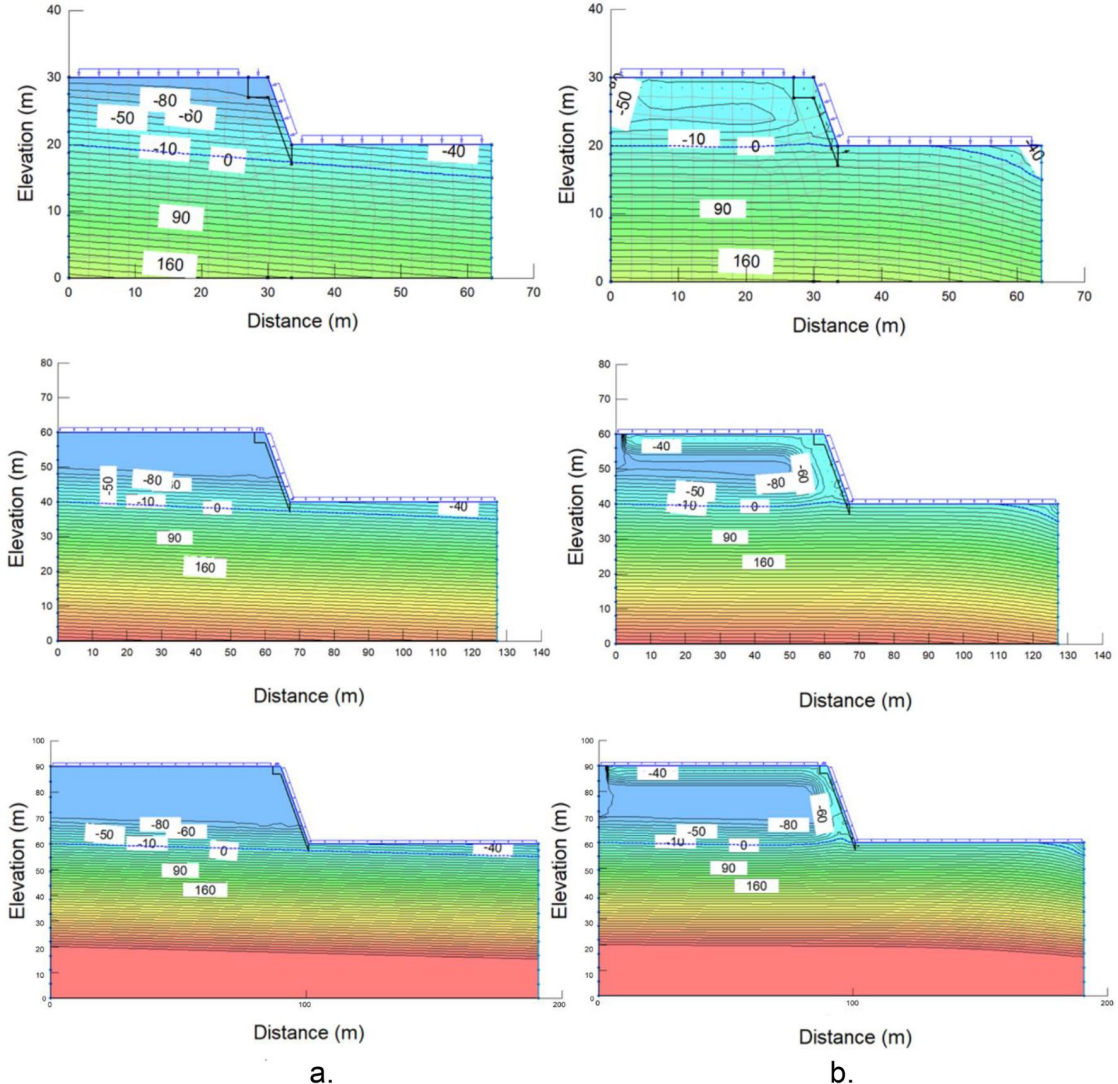
Pore-water pressure contours (**a**) at t = 0 day (before rainfall started) and (**b**) at t = 12 day (at the end of rainfall) for slope angle = 70° and heights = 10 m (top); 20 m (middle); 30 m (bottom).

Pore-water pressure profiles and contours were obtained from the results of seepage analyses using SEEP/W. Figure 10 displays the pore-water pressure contours for analysis of the effect of slope angle on the movement of infiltrated water. Figure 11 shows the results from the analysis of the height influence on rainwater infiltration within the investigated slope. In all cases, the water table rises only near the toe of the slope during the wet period. Figures 10 and 11 also demonstrate that the suction only decreased marginally near the ground surface. It shows that steel slag lessens rainwater infiltration into the slope, regardless of slope height and angle.

**Figure 12:**
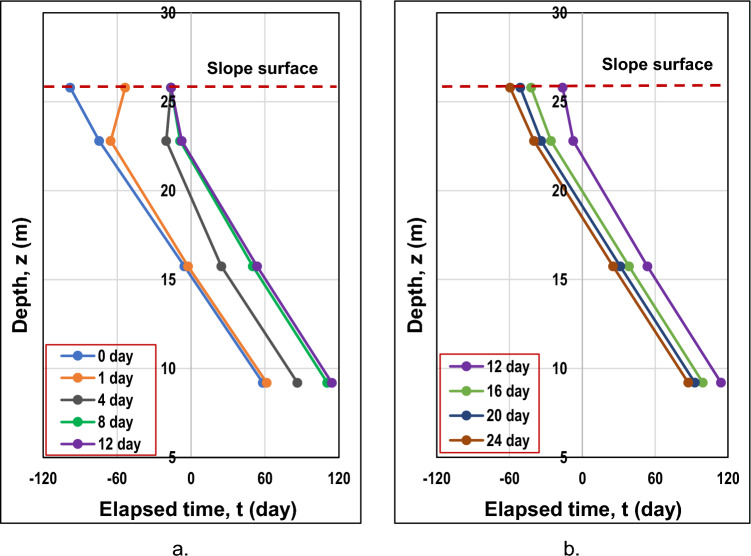
Pore-water pressure profiles during rainfall (**a**) and dry periods (**b**) at the middle of a slope with a height of 10 m and an angle of 27°

Figure 12 demonstrates one example of pore-water pressure profiles from seepage analyses for wet and dry periods. These curves can provide information about the pore-water pressure difference between the day before rainfall started (day 0) and at the end of rainfall (day 12). For steeper slopes, at a depth close to the slope surface, the pore-water pressure difference between days 0 and 12 is more significant than for gentler slopes but lower at a greater depth.

**Figure 13:**
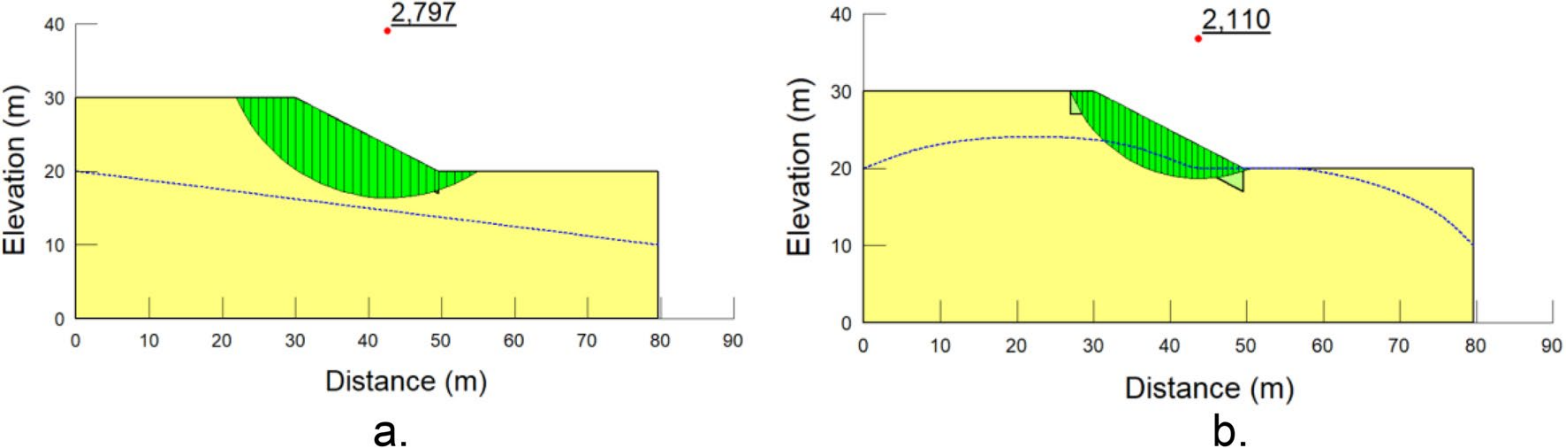
Critical slip surface at (**a**) t = 0-day (before rainfall started) and (**b**) t = 12 day (at the end of rainfall) for slope height = 10 m and slope angle = 27^o^.

Based on the analyses using SLOPE/W, the factor of safety versus time curves were generated. Moreover, based on the grid and radius, it produces several slip surfaces, including the critical one. For example, Fig. 13 exhibits critical slip surfaces for days 0 and 12. It is clearly seen that the factor of safety is high enough, although it decreased from 2.797 to 2.110.

**Figure 14:**
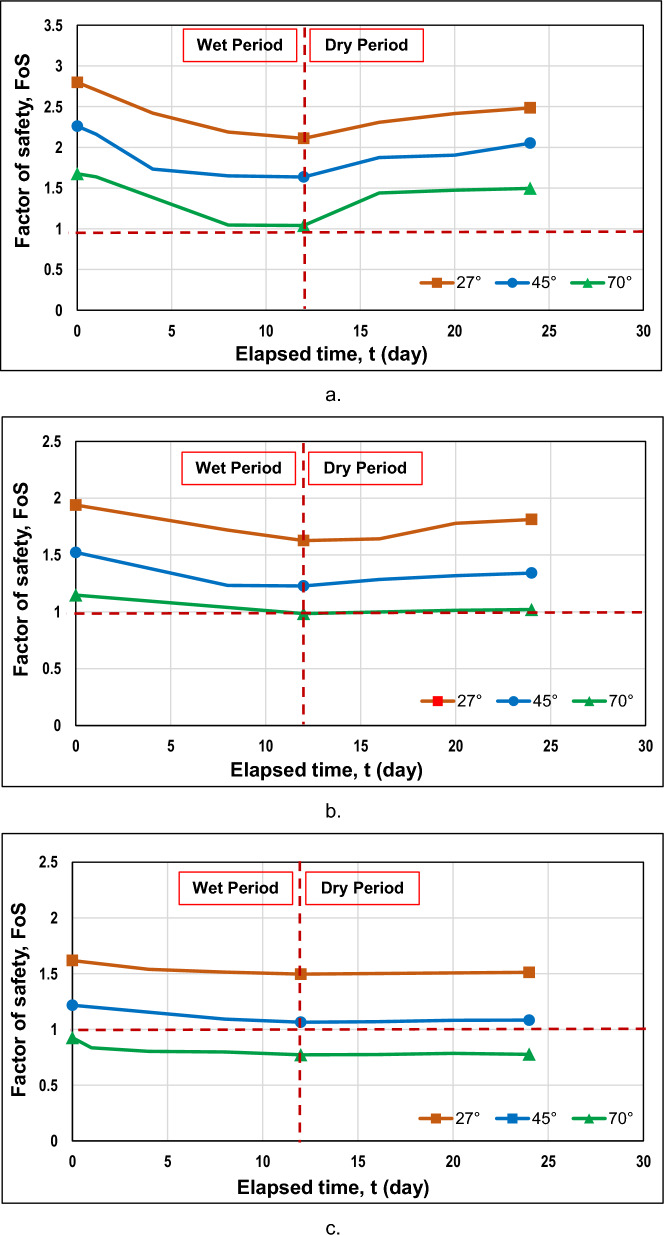
The factor of safety variations for (**a**) 10 m, (**b**) 20 m, and (**c**) 30 m heights.

During the wet period, the safety factor should gradually decrease until the end of the rainfall duration and then slowly increase to become steady. Figure 14 represents the graphs of a safety factor versus time derived from nine sets of analyses for three different heights (10 m, 20 m, and 30 m) and three different angles (27°, 45°, and 70°). Most of the curves distinctly demonstrated the trend described above, since until day 12, a factor of safety for almost all the cases underwent a downward trend and afterwards started to increase slowly. The reason for this particular response lies in the effect of rainfall on soil suction. During the rainfall period, soil suction decreases, reducing the shear strength of the soil; however, once the rainfall ends, it gradually starts to increase again since the drying process will initiate.

Another tendency derived from the graphical data is the inverse relationship between height and safety factor. Higher slopes negatively affect the safety factor. Moreover, gentler slopes tended to have a greater safety factor than steeper slopes, which had lower values. Specifically, for a gentle slope, the factor of safety dropped from 2.80 to 1.62; in the meantime, for a steeper slope, the factor of safety reduced from 1.68 to 0.93, where the first values are related to the lowest slope. The worst scenario out of these trials was the steepest and highest slope, as the factor of safety was below 1. Figure 14 illustrates that for slopes inclined at an angle of 70°, it can be stated that the factor of safety did not meet the minimum criterion since the values were found near or below 1. Apart from that, the steepest and lowest slope had a factor of safety close to 1. It indicates that steel slag is not applicable as a cover for steeper slopes.

For the purpose of determining the effect of the steel slag on the slope response, it is essential to provide cases without remediation. Taking into consideration the results shown in Fig. 14a–c, slopes with heights of 30 m and slope angles of 70° were omitted from the analyses of slopes with original soil only. It is critical to note the effective friction angle of steel slag, which is high enough and equal to 42°. The relationship between shear strength and effective friction angle can be derived from Eq. (4), where higher values of friction angle suggest greater shear strength. However, there is a dependency on the slope's steepness, and when steeper slopes are examined, the shear strength will be exceeded by shear stress, which will trigger the failure of a slope. Hence, four more analyses for slopes with original soil only were considered, and the results were compared with the steel slag-covered slopes.

**Figure 15:**
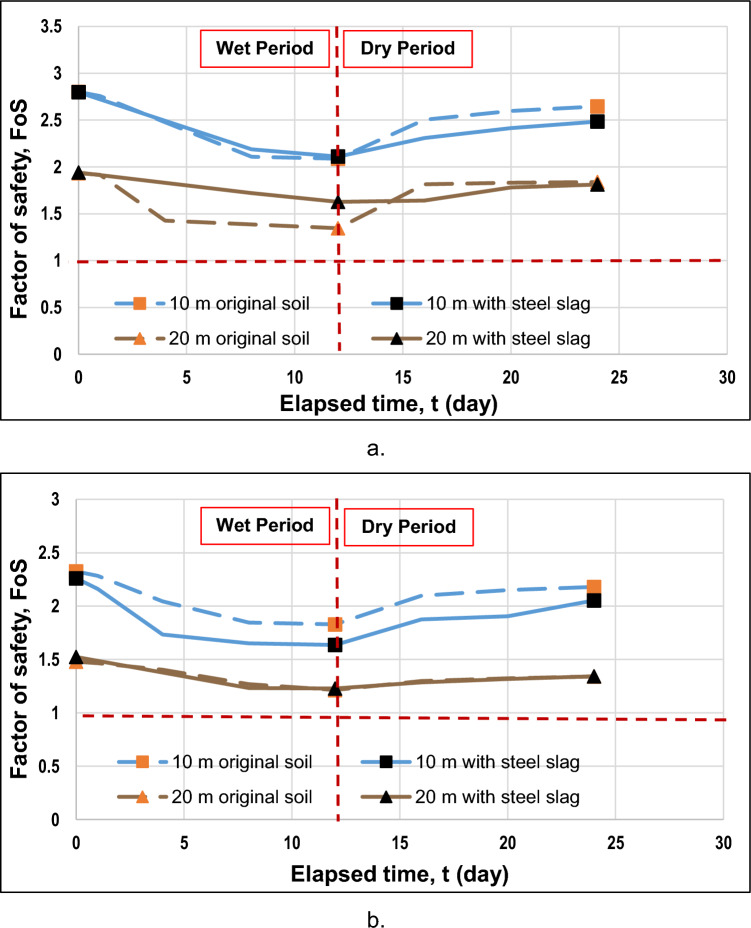
The comparison of the factor of safety against the time for a slope with and without steel slag when slope angles are (**a**) 27° and (**b**) 45°

Figure 15a provides a comparison between the factor of safety of slopes with and without steel slag. It can be noted that for a slope angle of 27°, steel slag increases the factor of safety during the wet period; nevertheless, the factor of safety appeared lower than that of the original slope after the end of the rainfall because steel slag retained the infiltrated water. Furthermore, for both slope heights, the initial factors of safety were nearly the same. When a 20-m slope height was examined, the factor of safety at the end of rainfall for a steel slag-covered slope was higher (1.627) than that of the original slope (1.346); for a 10-m slope height, they were relatively the same. Additionally, the factor of safety difference was lower for slopes with steel slag (i.e., 0.313 < 0.588 for a 20-m slope height); moreover, the curves were more constant, especially when a slope height was 20 m. The reason why the original slopes had higher factor of safety difference values is due to the dynamic nature of the wet period, where a less steady state prevails when rainfall infiltrates the soil. However, this was not the case when the slope was covered with the steel slag, as it maintained the slope's stability by minimizing rainfall infiltration.

According to Fig. 15b, when a slope of 45° inclination was considered, the factor of safety with steel slag appeared to be lower or almost the same for 10-m and 20-m slope heights, respectively, which suggests that steel slag is effective only for gentler slopes. Furthermore, Fig. 15 reveals that the greater difference in factor of safety was observed for the 10 m-high slopes. One of the reasons why it had occurred was presumably due to the geometry of a slope, a less rapid water infiltration rate, and the capacity of water to runoff at a fast pace on steeper slopes. It can be explained through soil suction. The more raindrops that are infiltrated into the soil, the more it will reduce soil suction. Due to the reduction in soil suction, the factor of safety will also decrease since the soil will become weaker. For higher slopes, the infiltrated water will not penetrate as deeply as compared to lower heights for this duration. The suction will not have a substantial decrease, as there is less infiltrated rainwater on steeper and higher slopes.

## Conclusions

Application of steel slag is common for various construction activities; however, it is very limited in the geotechnical area, especially with regard to slope stability problems using unsaturated soil mechanics. The main objective of this study was to incorporate steel slag as a slope cover to promote sustainability and ensure slope stability under rainfall conditions for different slope geometries. Since the Almaty region is susceptible to landslides, the utilization of steel slag for slope stabilization is found to be a favourable preventive measure. According to the obtained results, steel slag had the same properties as natural aggregate or gravel, which confirms that it is a sustainable waste material that can be utilized in slope stabilization instead of natural coarse-grained materials. Based on slope stability analysis results, it was observed that there was a little difference between initial and final safety factor values for 20 and 30 m slope heights during both wet and dry periods. Even though the differences in safety factors for high and steep slopes during rainfall were relatively constant, slopes of 70° inclination are impossible to construct if the height of the slope is between 20 and 30 m, as the corresponding safety factor values were below the required minimum. The differences in factor of safety for slopes covered with steel slag during rainfall are fairly consistent and lower than those of original slopes since the steel slag helps to reduce water infiltration into the soil layer below it. Therefore, it can be deduced that the steel slag can be used as an effective slope cover to maintain the stability of the gentler slopes within 10–20 m. However, it is recommended to examine environmental aspects, including the possible leaching of toxic heavy metals. Moreover, this study has potential limitations. The study is mainly focused on the short-term effects of steel slag as a slope cover during and after rainfall events. Long-term observations and field investigations could help gain a better knowledge of steel slag's long-term performance in slope stabilization under different environmental conditions. The current slope profiles are too simplified. In practice, soil deposition and stratification can impact the variability of materials. In the future, it is expected to consider non-uniform soil profiles to make the analysis more credible.

## Data Availability

The datasets used and/or analysed during the current study available from the corresponding author on reasonable request.
